# Interaction between TRPML1 and p62 in Regulating Autophagosome-Lysosome Fusion and Impeding Neuroaxonal Dystrophy in Alzheimer's Disease

**DOI:** 10.1155/2022/8096009

**Published:** 2022-01-25

**Authors:** Lu Zhang, Yu Fang, Xuan Cheng, Yajun Lian, Hongliang Xu

**Affiliations:** ^1^Department of Neurology, The First Affiliated Hospital of Zhengzhou University, Zhengzhou 450052, China; ^2^Department of ICU, The First Affiliated Hospital of Zhengzhou University, Zhengzhou 450052, China

## Abstract

The loss of transient receptor potential mucolipin 1 (TRPML1), an endosomal and lysosomal Ca^2+^-releasing channel, has been implicated in neurodegenerative disorders. Mounting evidence have shown that TRPML1 could clear intraneuronal amyloid-*β* (A*β*), which triggers a hypothesis that TRPML1 activation may be beneficial for axonal transport in Alzheimer's disease (AD). In this work, the functional roles of TRPML1 were studied in the APP/PS1 transgenic mice and A*β*1-42-stimulated hippocampal neurons HT22. We found that lentivirus-mediated overexpression of TRPML1 was shown to promote an accumulation of autolysosomes and increase brain-derived neurotrophic factor (BDNF) transportation to the nucleus, suggesting an axon-protective function. More importantly, we found that TRPML1 also increased p62 that interacted with dynein. Lentivirus-mediated knockdown of p62 or inhibition of dynein by ciliobrevin D stimulation was found to reduce autolysosome formation and nuclear accumulation of BDNF in HT22 cells with A*β*1-42 stimulation. Inhibition of p62 by XRK3F2 stimulation was observed to promote the death of hippocampal neurons of the APP/PS1 transgenic mice. TRPML1 recruited dynein by interacting with p62 to promote the autophagosome-lysosome fusion to mediate BDNF nuclear translocation to impede axon dystrophy in mice with Alzheimer-like phenotypes. In summary, these results demonstrate the presence of a TRPML1/p62/dynein regulatory network in AD, and activation of TRPML1 is required for axon protection to prevent neuroaxonal dystrophy.

## 1. Introduction

Alzheimer's disease (AD), the commonest cause of dementia, is still considered as one of the greatest challenges in public health of the 21^st^ century [1]. AD is characterized by the presence of two key hallmark findings in pathology: neurofibrillary tangles and senile plaques, along with other dominant pathological changes such as neuronal loss and dystrophic neurites [2]. During the natural cause of ageing, selective intraneuronal amyloid-*β* (A*β*) accumulation and oligomerization in adult life may potentially lead to the degeneration of basal forebrain cholinergic neurons in AD [3]. A*β*-driven neurodegeneration begins in the axons, whereby protecting the axon may provide a new insight in the prevention of neuronal death [4]. In addition, brain-derived neurotrophic factor- (BDNF-) regulated tropomyosin-related kinase B (TrkB) retrograde axon transport is damaged in AD transgenic mouse neurons, while A*β* oligomers contributed to the deficit in axon transport [5]. Recently, the neurodegeneration in AD has been extended from the proposed idea of neuronal loss and astrogliosis to alterations in the early stage of AD such as synaptic and dendritic impairments and disorders in adult neurogenesis, including aberrant innervation [6].

The lysosomal channel transient receptor potential mucolipin 1 (TRPML1) is responsible for maintaining low pH and calcium levels that is critical for lysosomal function [7]. Additionally, loss-of-function of TRPML1 is closely related to neurodegeneration and accumulation of autophagy [8]. Autophagy is an essential cellular process which eliminates molecules and subcellular elements through lysosome-mediated degradation, thus promoting homeostasis, differentiation, development, and survival; autophagy is intimately related to aging as well as disease development and progression [9]. Chaperone-mediated autophagy loss has synergistic negative effects on the proteome at risk of aggregation in a mouse model of AD, thereby enhancing neuronal disease vulnerability and promoting the progression of AD [10]. Additionally, a recent study has reported that mitophagy diminishes insoluble A*β* and A*β* and prevents cognitive impairment in models with Alzheimer-like alterations through microglial phagocytosis of extracellular A*β* plaques and suppression of neuroinflammation [11]. A TRPML1 agonist has been found to induce the efflux of calcium, acidification of luminal, clearance of sphingomyelin, and A*β* in lysosomes in the transgenic gp120/APP/PS1 mice following infection by human immunodeficiency virus in the brain [12]. More specifically, TRPML1 upregulation also attenuated the A*β*1-42-blocked mammalian target of rapamycin (mTOR)/S6K regulatory axis and the expression of autophagic lysosome-reformation-associated proteins induced by A*β*1-42 in primary neurons isolated from APP/PS1 transgenic mice [13]. Notably, in TRPML1^−/−^ mouse neurons, light chain 3- (LC3-) positive autophagosomal puncta increased, which indicated elevated autophagosome formation and impaired autolysosome formation [14].

Furthermore, protein inclusions in neurodegenerative diseases are correlated to p62, which has an important function in the autophagic clearance of polyubiquitinated proteins [15]. p62 is required for appropriate dynein motility and trafficking along microtubules [16]. Therefore, the dysfunction of dynein caused neuron swelling and neurofilament accumulation, and modified neuronal activity through endocytic disturbances may trigger age-associated damage of cognitive functions [17]. In the present study, we thus developed *in vivo* and *in vitro* models in APP/PS1 double transgenic mice and hippocampal neurons with Alzheimer-like alterations to investigate regulatory network and function of TRPML1 in neuroaxonal dystrophies (NAD), autolysosome formation, and cognitive deficits during AD process.

## 2. Materials and Methods

### 2.1. Development of Mouse Model with Alzheimer-Like Phenotypes

In the animal experiments, we used 108 male APP/PS1 double transgenic mice with Alzheimer-like phenotypes, and 36 C57BL/6J wild-type (WT) male mice (aged 3 months/12 weeks, weighing 20-25 g) were purchased from Nanjing Animal Research Center of Nanjing University (Jiangsu, China). They were raised in a specific pathogen-free environment under a 12 h artificial light/darkness cycle, with water and food taken freely. A progressive increase of *β*-amyloid peptide deposition was revealed, and cellular immunologic activity was detected in some brain areas when mice were 3-4 months of age. By 6 months, synaptic transmission and long-term polarization were significantly impaired. Conformational changes and hyperphosphorylation of tau protein aggregates in the hippocampal tissues were detected in the mice (12–15 months old), revealing synaptic dysfunction-related plaques and neurofibrillary tangles, which are similar to the phenotypes observed in patients with AD [18].

TRPML1-overexpressed APP/PS1 transgenic mice were constructed, as previously described, by the injection of adenovirus harboring TRPML1 [19–21]. APP/PS1 transgenic mice were classified into 4 subgroups in a random manner: WT group, APP/PS1 group (untreated APP/PS1 mice with Alzheimer-like phenotypes), APP/PS1/TRPML1^+/+^ group (APP/PS1 mice with Alzheimer-like phenotypes were treated with TRPML1^+/+^), and APP/PS1/TRPML1^+/+^+XRK3F2 group (APP/PS1 mice with Alzheimer-like phenotypes were treated with TRPML1^+/+^ alone or combined with p62 inhibitor XRK3F2).

Briefly, 3-month-old mice with Alzheimer-like phenotypes and WT mice were subjected to 1% pentobarbital sodium injection intraperitoneally (50 mg/kg), followed by injection of adenovirus with overexpressed TRPML1 (Ribo Biotechnology Co., Ltd., Guangzhou, China; 1.5 *μ*L; viral titer of 1.2 × 10^9^ vg/mL) and p62 inhibitor XRK3F2 (40 mg/kg/day; CAS 2375193-43-2; ChemGen Corp., Gaithersburg, MD) (0.2 *μ*L/min) in the bilateral hippocampus (2.3 mm anterior to the skull; 1.8 mm; depth, 2.0 mm). Then, the expression of TRPML1 in the hippocampal tissues was detected 1, 3, and 6 months after injection. There were 12 mice in each subgroup. The hippocampal brain tissues were collected for brain homogenate, and the other tissues were prepared for paraffin sections, after the tissues were perfused with anesthesia and phosphate-buffered saline (PBS). The animal experiment protocols were strictly carried out under the Guide for the Care and Use of Laboratory Animals of the National Institutes of Health and under the approval of the Ethics Committee of the First Affiliated Hospital of Zhengzhou University.

### 2.2. Golgi-Cox Staining

This staining method is one of the most efficient techniques to study morphological changes in normal and abnormal neurons. Communication between neurons usually occurs in the synapse while synapse loss and dysfunction are considered as the mechanism underlying AD-induced cognitive impairments. As the main part of the postsynaptic element, the dendritic spine is a major postsynaptic site responsible for neuroexcitatory neurotransmission. Morphological changes of neurons in a model of AD are usually accompanied with abnormal changes of the dendritic spine.

The FD Rapid Golgi Stain Kit (PK4O1/PK401A, FD NeuroTechnologies Inc., Columbia, MD) was employed to stain mouse hippocampal tissues. Mouse brains were soaked at room temperature in the Golgi-Cox staining solution (A plus B) for 2 weeks, and then, at 4°C in solution C for 72 h, which were kept in the dark. Sections were later sliced into 100 *μ*m thick sections in a cryostat at -22°C, which were further left to stain with solutions D and E for 15 min and dehydrated. Dendrites and dendritic spines from 3 to 4 dendrites in each neuron were observed under a microscope (Wetzlar, Germany). Lastly, the calculation on the dendritic spine density was employed in a blind manner according to the formula: the dendritic spine density = the number of spines/the length of dendrites.

### 2.3. Congo Red Staining

According to the experimental details described in an earlier literature [22], we observed the formation of senile plaques in hippocampal tissues by Congo red staining. After routine deparaffinization and hydration, the hippocampal tissue sections were left to stain in the solutions of Gill's hematoxylin (Sigma, St. Louis, MO), alkaline sodium chloride, and Congo red (0.2% saturated sodium chloride in 80% ethanol; Sigma) for 10 min, 20 min, and 15 min, respectively. Finally, after dehydration in 95% alcohol solution, the sections were microscopically observed.

### 2.4. Morris Water Maze (MWM) Test

We conducted the MWM test to evaluate the spatial learning and spatial memory abilities of the experiment mice (*n* = 10 per group). The MWM test was composed of a white-opaque water-filled black circular tank (120 cm in diameter, 60 cm in height, and 45 cm in depth), and the water temperature was 20°C-22°C. In order to make sure the platform was invisible to mice, the nontoxic black pigment was added to make the tank opaque. Following this, the water tank was separated into four imaginary quadrants. At 1 cm below the water surface in the target quadrant middle, we placed a round transparent platform (8 cm in diameter). During the test of place navigation, the mice were released once in every start position (four in total) every day for five days. Then, in order to measure the escape latency, we allowed the mice to locate the platform to record the finding time. The time of searching platform for each mouse was no more than 60 s. Meanwhile, we guided the mouse failed to locate the platform within 60 s toward the platform and kept it stay on the platform for 10 s. In this event, we recorded the escape latency as 60 s. After that, on day 6 (24 h post the last test of escape latency), the probe test was employed. Subsequently, after the removal of the platform, we allowed the mice to search the escape platform also within the time no more than 60 s. The recording on the escape latency and times of passing through the platform was performed with the use of an ANY-maze Behavior Analysis System.

### 2.5. Cell Apoptosis Evaluation

For elimination of endogenous peroxidase (POD), five dewaxed sections added with 50 *μ*L of 1% protease K diluent for 30 min were treated using 0.3% H_2_O_2_ methanol solution for 30 min at 37°C. These sections were then subjected to 1 h incubation at 37°C using the TUNEL solution in a humidified chamber in the dark. Subsequently, after further 30 min incubation in 50 *μ*L of converter-POD at 37°C, the sections were allowed to develop with 2% diaminobenzidine at room temperature for 15 min. Following this, after hematoxylin counterstaining, the sections were microscopically observed. Ten randomly selected visual fields of each section were used to count the TUNEL-positive cells and normal cells, and the rate of apoptotic cells was calculated.

### 2.6. RNA Isolation and Quantification

Trizol reagent (Invitrogen Inc., Carlsbad, CA) was applied for total RNA extraction from tissues and cells, followed by concentration determination by NanoDrop 2000 software (Thermo). Then, reverse transcription (RT) protocol was carried out with the PrimeScript™ RT reagent kit with the gDNA Eraser kit (RRO37A, Takara Holdings Inc., Kyoto, Japan). Next, real-time quantitative polymerase chain reaction (qPCR) was employed with the use of the SYBR® Premix Ex Taq™ (Tli RNaseH Plus) kit (RR820A, Takara) combined with ABI7500 qPCR instrument (Thermo Fisher Scientific Inc., Waltham, MA). The relative quantification value for the target genes normalized to the expression pattern of glyceraldehyde-3-phosphate dehydrogenase (GAPDH) was identified by the 2^-*ΔΔ*Ct^ method [23]. All used primers provided by GenePharma Co., Ltd., (Shanghai, China) are shown in Table S1.

### 2.7. Protein Extraction and Quantification

Protease inhibitor-contained phenylmethanesulfonyl fluoride was applied for extracting the total protein, and the concentration was identified by the bicinchoninic acid kit (23227, Thermo Fisher Scientific). Next, equal amounts of protein were separated and transferred by the electrophoretic transfer method to polyvinylidene fluoride membranes, followed by 1 h incubation using 5% bovine serum albumin (BSA). Following this, the membrane was subsequently incubated overnight at 4°C with the following primary antibodies: TRPML1 (1 : 200, ab28508, Abcam Inc., Cambridge, UK), brain-derived neurotrophic factor (BDNF) (1 : 100, ab203573, Abcam), phosphorylated cAMP-response element-binding protein (p-CREB) (1 : 5000, ab32096, Abcam), CREB (1 : 1000, ab32515, Abcam), p-tyrosine kinase receptor B (p-TrkB) (1 : 100, ab109684, Abcam), TrkB (1 : 200, ab18987, Abcam), p-dynein (1: 2000, ab11244, Abcam), dynein (1 : 1000, A3624, ABclonal, China), p62 (1 : 1000, A19700, ABclonal), and GAPDH (1 : 5000, ab8245, Abcam). The next day, the membrane was subjected to further 1.5 h incubation at room temperature with the horseradish peroxidase-coupled secondary antibody goat anti-rabbit immunoglobulin G (IgG) (1 : 20000, ab205718). The protein bands were developed and visualized by chemiluminescence reagent (NCI4106, Pierce, Rockford, IL) on an ImageJ 1.48u software (Bio-Rad, Hercules, CA). As normalized to the gray value of the GAPDH band, the gray value of the target protein band was quantified, which was regarded as the relative expression of the target proteins.

### 2.8. Cell Culture and Infection

The isolation and treatment of primary hippocampal neurons were carried out as described previously [24]. The morphology of hippocampal neurons was observed under an inverted microscope. The pregnant C57BL/6J mice on E17.5-E18.5 days, postanesthesia, were placed under an anatomy microscope. The anatomical area was disinfected by alcohol. Fetal mice were rapidly removed through V-shaped laparotomy and placed in a precold isotonic buffer. The brains of fetal mice were immediately isolated while the surrounding blood vessels and fibrous tissues were removed. The cortical areas were dissected microscopically and rapidly moved into the Dulbecco's modified Eagle's medium (DMEM) (Gibco, Grand Island, NY). Next, the cortical tissues were quickly cut into pieces, centrifuged for 3 min at 1000 rpm, and detached in water bath at 37°C supplemented with papain (2.5 U/mL) and DNase (250 U/mL) over gentle shaking every 5 min. After filtering through a 200-mesh filter, centrifugation was performed at room temperature at 100 rpm for 5 min, with 10% fetal bovine serum- (FBS-) contained DMEM subsequently added in. After that, the primary cortical cells were counted using a cell counting plate and implanted in a 6-well plate coated with polylysine (1 × 10^5^), followed by renewal of medium 4 h later. Following the addition of neural differentiation medium, half of medium was further renewed every 3 days. The hippocampal neurons were transferred into a 96-well plate (1 × 10^5^ cells/well) after 6 days, followed by 10 *μ*M of A*β*1-42 treatment after adhesion.

Besides, hippocampal neuron HT22 cells (Procell Life Science & Technology Co., Ltd., Wuhan, Hubei, China) were maintained in 10% FBS and 1% penicillin-streptomycin-supplemented DMEM and cultured at 37°C in 5% CO_2_. After being trypsinized with 0.25% trypsin, the cells were passaged at a ratio of 1 : 3, which were then seeded into a 6-well plate (3 × 10^5^ cells/well) and harvested for following experiments when cells were at the exponential growth phase.

TRPML1 antagonist (SB705498; 10 nM, S2773, Selleck) and activator (capsaicin; 50 nM, S1990, Selleck) were incubated with HT22 cells for 12 h, and relevant detection was conducted. The recombinant lentivirus (GenePharma) overexpressing the TRPML1 gene (oe-TRPML1) and short hairpin RNA (shRNA) against TRPML1 (sh-TRPML1) were constructed by the amplification of the TRPML1 gene. After infection and purification, the expression patterns of TRPML1 were evaluated.

### 2.9. Detection of Lysosomes and Autolysosomes

Neurons were infected with adenovirus harboring 5 × 10^10 m^ PFU/mL of mCherry-GFP-LC3B (Hanbio Co., Ltd., Shanghai, China), and autolysosomes were labeled with mRFP-GFP-LC3. Cells were deprived of nutrients and incubated in a serum-deprivation medium with NaCl (140 mM), CaCl_2_ (1 mM), MgCl_2_ (1 mM), glucose (5 mM), and HEPES (20 mM; pH = 7.4) at 37°C. MRFP-GFP-LC3 autophagy double standard adenoviruses (Hanbio) were placed on ice, diluted by medium, and used to infect hippocampal neurons. After 48-72 h, cells were fixed, mounted, and photographed. Bafilomycin A1 (10 nM, 30 nM, and 50 nM, AbMole BioScience Inc., Houston, TX) was used to block autophagy flux. Observation was performed after 48 h of infection.

### 2.10. Immunofluorescence

For immunofluorescence analysis, cells were left to fix in 4% paraformaldehyde for 10-15 min and blocked in BSA- (3 mg/mL) contained 0.25% Triton X-100 for 30 min. After overnight incubation with primary antibodies at 4°C, including anti-BDNF (ab108319; Abcam) and anti-Sternberger monoclonals were left to incorporate (anti-SMI-31, Covance, Princeton, NJ), the cells were subsequently allowed to incubate at room temperature using fluorophore-conjugated secondary antibodies with Alexa Fluor® 594 (1 : 200, AB150088; Abcam) or Alexa Fluor® 488 (1 : 200, AB150081; Abcam) for 1 h. Then, DAPI counterstaining was applied for cell nuclear staining, followed by microscopic observation under a fluorescent microscope (Zeiss, Thornwood, NY) or a FV1000 confocal microscope [25].

### 2.11. Detection of BDNF Nuclear Translocation

The cytoplasmic and nuclear proteins were extracted from cells and tissues using a cytoplasm and nuclear protein extraction kit (P0027, Beyotime Biotechnology Co., Ltd., Shanghai, China). Following this, the cells were placed in low osmotic pressure conditions and allowed to expand until cell membrane ruptured, to allow cytoplasmic protein to be released; nuclear precipitation was later obtained by centrifugation. Finally, the nuclear protein was isolated with a high-salt nuclear protein extraction reagent to detect the BDNF expression in the nucleus. Immunofluorescence staining with antibodies to BDNF was performed on tissues and cells. After routine deparaffinization and hydration of tissues, antigen retrieval was performed, and the tissues were blocked at room temperature in BSA for 30 min. The cells were blocked with 3 mg/mL BSA, 100 mM glycine, and 0.25% Triton X-100 in PBS for 30 min. After that, the tissue and cell samples were subjected to overnight incubation with primary antibodies and then with fluorescein isothiocyanate- (FITC-) labeled secondary antibody (ab6717, Abcam). DAPI was applied for nuclear staining. After another 3 washes with PBS, the amount of BDNF in the nucleus was observed under a laser confocal microscope and quantitatively analyzed.

### 2.12. Detection of Perinuclear Distribution of Lysosomes

Neuronal cells plated in 6-well plates were added with Lamp1-mCherry fluorescent protein adenovirus (5 × 10^10^ PFU/mL) after the cells adhered. Afterwards, the expression of cell fluorescence was microscopically observed from 24 h to 48 h. In order to label lysosomes, Lamp1-mCherry fluorescent protein adenoviruses were used to infect hippocampal neurons. The perinuclear distribution of lysosomes was observed under a confocal microscope and analyzed by ImageJ.

### 2.13. Colocalization of TRPML1 and Lysosomes

After 24 h, 4% paraformaldehyde was applied for fixation of the HT22 cells seeded in 6-well plates for 10 min, followed by permeabilization at room temperature for 10 min using 0.5% Trion-X 100. Cells were then left to block at room temperature with goat serum for 1 h and subsequently probed at 4°C overnight with primary antibodies to TRPML1 (ab272608, Abcam) and lysosome-labeled antibodies to LAMP1 (ab24170, Abcam). Following 3 washes with PBS Tween-20 (PBST), corresponding fluorescent secondary antibodies were added at room temperature for an additional 1 h incubation. DAPI was lastly added for staining, and the glycerol-mounted sections were subjected to microscopic observation.

### 2.14. Coimmunoprecipitation (Co-IP) Assay

The cells were lysed with ice-cold lysis buffer containing 150 mM NaCl, 50 mM Tris-Cl (pH = 7.4), 1 mM ethylenediaminetetraacetic acid, and 1% Triton. Cellular debris was removed by centrifugation. Whole-cell lysates (input) were collected, and every 500 *μ*g was incubated with antibodies against TRPML1 (1 : 100, ab272608, Abcam) and dynein (1 : 100, ab156567, Abcam) at 4°C. Dynabeads protein G (Invitrogen Inc., Carlsbad, CA) was used to pull down protein complexes. IgG (Santa Cruz Biotechnology Inc., Santa Cruz, CA) was the antibody used in negative control (NC) samples. The beads were then washed 4 times with ice-cold lysis buffer, and the protein was eluted in 1x sodium dodecyl sulfate (SDS) loading buffer. Finally, Western blot analysis was performed to examine the protein interactions [26].

### 2.15. Immunocytochemistry

Neurons were seeded in Petri dishes (1 × 10^6^ cells/mL), and three coverslips were spread in the Petri dish and cultured for 5 days. Following 20 min fixation in 4% paraformaldehyde (30525-89-4, Sigma-Aldrich Chemical Company, St. Louis, MO), the cells were incubated using 0.5% Triton X-100 (T9284MSDS, Sigma-Aldrich) for 20 min, 3% H_2_O_2_ for 15 min, and blocked with serum for 20 min. After cells were probed with the primary antibodies to TRPML1 (1 : 100, ab28508, Abcam) at 37°C for 1 h, the cells were visualized by DAB for 3-5 min followed by hematoxylin restaining and observed under a microscope.

### 2.16. Flow Cytometry

Neurons following treatment were centrifuged with the supernatant discarded. According to the instructions of the Annexin-V-FITC cell apoptosis detection kit (K201-100, BioVision, Milpitas, CA), Annexin-V-FITC, propidium iodide (PI), and HEPES buffer solution was mixed into Annexin-V-FITC/PI staining solution at the ratio of 1 : 2 : 50. The cells were resuspended to a final density of 1 × 10^6^ cells per 100 *μ*L of dye solution and mixed by shaking. After incubation at room temperature for 15 min, 1 mL of HEPES buffer solution was added to the cells and mixed. Fluorescence was initiated by excitation at 488 nm and was measured by emission filters at 515 nm (FITC) and 620 nm (PI).

### 2.17. Fluorescence Resonance Energy Transfer (FRET)

The p62-YFP (pEYFP-C1-p62) recombinant vector and the pCFP-TRPML1 recombinant vector were constructed. C57BL/6J mice aged 4–6 months were selected and anesthetized with injection of ketamine (100 mg/kg)/xylazine (10 mg/kg). Next, 100 *μ*g of endotoxin-free plasmid was diluted with normal saline (25 *μ*L), and the above two plasmids were slowly injected into mouse tibialis anterior muscle. pCFP-TRPML1 was used as the donor, and pEYFP-C1-p62 was used as the acceptor. The mice were grouped into pCFP-TRPML1+pEYFP-C1-control (the two plasmids pCFP-TRPML1 and pEYFP-C1 vectors were slowly injected into the mouse tibial anterior muscle) and pCFP-TRPML1+pEYFP-C1-p62 groups (the two plasmids pCFP-TRPML1 and pEYFP-C1-p62 were slowly injected into the mouse tibial anterior muscle). The sample was excited using a 458 nm laser and scanned in the range of 475-600 nm, after which detection was performed with Electropulsator ECM-830 (BTX, Holliston, MA). When TRPML1 interacted with p62 and the spatial distance was less than 10 nm, the fluorescence emitted by the donor after being excited cannot be detected in the tissue, but only the yellow fluorescence emitted by the excited acceptor can be detected.

### 2.18. Bioinformatics Analysis

The keyword “Alzheimer's disease” was searched through the Gene Expression Omnibus database to obtain the AD-related dataset GSE5281. The samples in GSE5281 came from the human brain entorhinal cortex, including 13 control samples and 10 AD samples. The probe IDs of the expression profiles were converted to gene symbol using the platform annotation file GPL570 pair. Based on the R language “limma” package, the analysis of gene differential expression was conducted, with |log2FoldChange | >1 and *p* < 0.05 as the differential gene screening criteria. Also, a heat map of differentially expressed genes was plotted with the use of the R language “pheatmap” package. Through the GeneCards database (score ≥ 7) and the DisGeNet database, the keyword “Alzheimer's disease” was searched to obtain genes related to AD. To screen for key factors that alleviate AD, the jvenn tool was applied to obtain the intersection between the differentially underexpressed genes (top 300) and genes related to AD with the smallest *p* value.

To further predict the relevant genes of key factors, we calculated the Pearson correlation coefficients (correlation coefficient, Cor) of other genes and key factors based on GSE5281 microarray gene expression profiles with ∣Cor | >0.5 and *p* < 0.05 as the screening criteria. The top 2000 genes with the smallest *p* value and AD-related genes were selected using the jvenn tool to take an intersection to screen the factors. We employed the STRING tool to analyze the interaction relationships between candidate genes, and the relationship network was imported into Cytoscape 3.5.1 software for analysis to rank the degree (degree) of the genes.

### 2.19. Statistical Analyses

All statistical analyses were conducted in the SPSS 21.0 statistical software (IBM Corp., Armonk, NY). With normal distribution and variance homogeneity tested, the measurement data with normal distribution were expressed as the mean ± standard deviation. The significant difference between two groups was tested by the independent sample *t* test. Multigroup comparisons were conducted using one-way analysis of variance (ANOVA), followed by Tukey's multiple-comparisons tests. ANOVA of repeated measurements with Bonferroni's post hoc test was applied for multigroup comparisons at different time points. *p* value < 0.05 was considered a significant difference.

## 3. Results

### 3.1. Overexpression of TRPML1 Confers Axon Protection and Alleviates Cognitive Impairment in Mice with Alzheimer-Like Phenotypes

**Figure 1:**
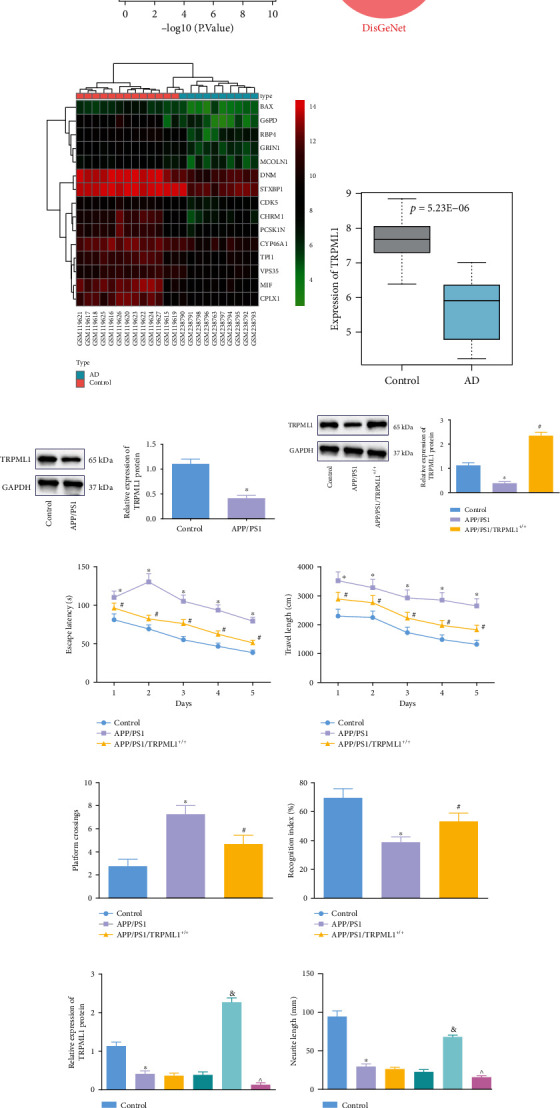
Overexpression of TRPML1 alleviates NAD and cognitive impairment in mice with Alzheimer-like phenotypes. (a) A volcano plot of differential gene expression analysis of the AD-related GSE5281 dataset. The abscissa represents -log10 (*p* value), the ordinate represents the logFC value, the red dots represent highly expressed genes, and the green dots represent poorly expressed genes. (b) Venn diagram of AD-related genes from the GeneCards and DisGeNet databases as well as differentially underexpressed genes. (c) A heat map of candidate gene expression in the AD-related GSE5281 dataset. The color scale from green to red indicates that the gene expression value is from low to high. (d) Expression of TRPML1 in AD samples (*n* = 10) and control samples (*n* = 13) in the AD-related GSE5281 dataset. (e) The protein expression of TRPML1 in the hippocampal tissues of APP/PS1 mice, determined by Western blot analysis. (f) The protein expression of TRPML1 in the hippocampal tissues of APP/PS1 mice in the absence or presence of TRPML1^+/+^, determined by Western blot analysis. (g) Escape latency of APP/PS1 mice in the absence or presence of TRPML1^+/+^. (h) The travel length of APP/PS1 mice in the absence or presence of TRPML1^+/+^. (i) Time crossing the platform of APP/PS1 mice in the absence or presence of TRPML1^+/+^. (j) Recognition indexes of APP/PS1 mice in the absence or presence of TRPML1^+/+^. (k) Expression of TRPML1 in primary neurons in response to A*β*1-42 alone or combined with oe-TRPML1/sh-TRPML1, determined by Western blot analysis. (l) Axonal length of neurons observed under an inverted microscope. Measurement data were described as the mean ± standard deviation. An unpaired *t-*test was used for comparison between the two groups. One-way ANOVA was used for comparisons among multiple groups, followed by Tukey's post hoc test. ANOVA of repeated measurements with Bonferroni's post hoc test was applied for multigroup comparisons at different time points. *n* = 12 for mice in each group. ^∗^*p* < 0.05 vs. C57BL/6J WT mice or untreated primary hippocampal neurons; ^#^*p* < 0.05 vs. APP/PS1 mice; ^^^*p* < 0.05 vs. primary hippocampal neurons treated with sh-NC+A*β*1-42; ^&^*p* < 0.05 vs. primary hippocampal neurons treated with oe-NC+A*β*1-42. The cell experiment was repeated 3 times independently.

Through differential gene expression analysis of the AD-related GSE5281 dataset, 3532 significantly differentially expressed genes were obtained, including 1650 highly expressed genes and 1882 poorly expressed genes (Figure 1(a)). Through the GeneCards database and DisGeNet database, 1659 and 3397 genes associated with AD were obtained, respectively. To screen the key factors that can alleviate the pathologies of AD, we took the intersection of the top 300 differentially underexpressed genes with the AD-related genes with the smallest *p* value to obtain 15 candidate genes (DNM1, CHRM1, GRIN1, MIF, RBP4, CYP46A1, STXBP1, G6PD, CDK5, TPI1, PCSK1N, CPLX1, BAX, VPS35, and MCOLN1) (Figure 1(b)) and further plotted the heat map of the screened candidate genes in microarray GSE5281 (Figure 1(c)). Then, the expression of TRPML1 protein (Alias: MCOLN1) was determined in APP/PS1 double transgenic mice. Moreover, the differential gene analysis results displayed a significantly low expression of TRPML1 in AD (Figure 1(d)).

Western blot analysis results also suggested a lower TRPML1 expression in the APP/PS1 mice relative to the C57BL/6J WT mice (Figure 1(e)). To verify how TRPML1 affects AD progression, we upregulated the TRPML1 expression in APP/PS1 mice (Figure 1(f)), followed by the MWM test for cognitive ability evaluation. It was found that the escape latency and travel duration required for APP/PS1 mice to cross the platform were reduced compared with that of normal mice, and the recognition indexes were improved while opposite results were found in the APP/PS1/TRPML1^+/+^ mice relative to the APP/PS1 mice (Figures 1(g)–1(j)). These results suggested that overexpression of TRPML1 presented a protective effect in mice with Alzheimer-like phenotypes.

Next, with the attempt to validate the influences of TRPML1 on axons in AD, TRPML1 was overexpressed or knocked down *in vitro* in the primary hippocampal neurons HT22 treated with A*β*1-42 (Figure 1(k)). To detect the effect of TRPML1 on axons, an inverted microscope was introduced to test the morphological changes of axons of primary hippocampal neurons in each group. In A*β*1-42-treated HT22 cells, axons showed bead-like appearances and were shortened, with small cell bodies, which were attenuated following overexpressing TRPML1 but aggravated following TRPML1 knockdown (Figure 1(l)). All these results suggested that TRPML1 exerted a protective effect on axons and attenuated cognitive impairment in mice with Alzheimer-like phenotypes.

### 3.2. Autophagosome-Lysosome Fusion Facilitates BDNF Nuclear Transport to Block NAD in AD

Autophagosomes, mainly involved in the transformation of cytoplasmic proteins and organelles, could provide nutrition and clear damaged proteins. Autophagosomes in neurons are formed at the distant ends of axons and fused with a lysosome in somatic cells through retrograde transportation. Although it has been implicated that defective neuron autophagy may be related to neurodegenerative disorders, neuron autophagy functions are still not well understood. Autophagosomes could enhance neuron complexity and protect against neurodegeneration through the retrograde transportation of TrkB receptor activated by BDNF [27]. p150^Glued^/dynactin-dependent transport of autophagosomes supplemented with TrkB requires their association with the endocytic adaptor AP-2, an essential protein complex previously considered to function exclusively in clathrin-mediated endocytosis. These data highlight a novel noncanonical function of AP-2 during retrograde transport of BDNF/TrkB-loaded neuronal autophagosomes, uncovering a causal relationship between autophagy and the BDNF/TrkB signaling [28].

**Figure 2:**
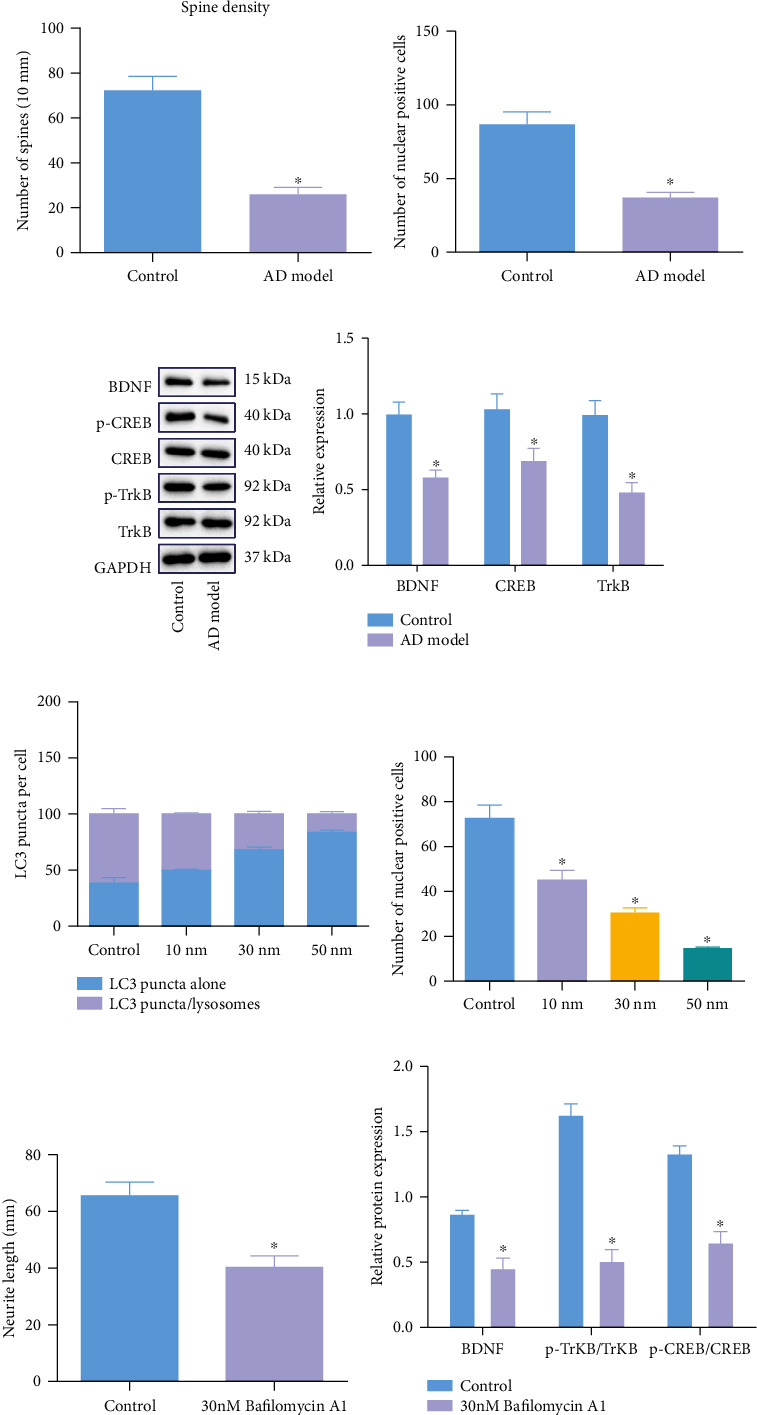
Autophagosome-lysosome fusion facilitates BDNF nuclear transport to block NAD in AD. (a) Morphological changes of neuron axons in the hippocampal tissues of mice. (b) Neuron nuclear expression of BDNF in the hippocampal tissues of mice. (c) Protein expression of BDNF, TrkB, and CREB, and the levels of TrkB and CREB phosphorylation extent in the hippocampal tissues of mice, assessed by Western blot analysis. (d) mRNA expression of BDNF, TrkB, and CREB in the hippocampal tissues of mice, evaluated by RT-qPCR. (e) Lysosome and autolysosome fusion in HT22 cells treated with Bafilomycin A1. (f) Changes of BDNF nuclear translocation in HT22 cells treated with Bafilomycin A1. (g) Length of axons in HT22 cells treated with Bafilomycin A1 (30 nM) for 24 h under an inverted microscope. (h) Protein expression of BDNF, TrkB, and CREB, and the levels of TrkB and CREB phosphorylation extent in HT22 cells after Bafilomycin A1 (30 nM) treatment. Measurement data were described as the mean ± standard deviation. The unpaired *t*-test was used for comparison between the two groups. One-way ANOVA was used for comparisons among multiple groups, followed by Tukey's post hoc test. The cell experiment was repeated 3 times independently. *n* = 12 for mice in each group. ^∗^*p* < 0.05 vs. C57BL/6J WT mice or untreated HT22 cells.

To validate the effect of autolysosome formation on BDNF nuclear translocation in AD, we assessed the morphology of axons in hippocampal regions of mice. Golgi-Cox staining results illustrated that APP/PS1 mice showed decreased dendritic spines in the hippocampal tissues compared with that of the control mice (Figure 2(a)) and C57BL/6J WT mice. The hippocampal tissues of APP/PS1 transgenic mice presented reduced BDNF nuclear translocation (Figure 2(b)), decreased mRNA expression of BDNF, and lower ratio of p-TrkB/TrkB and p-CREB/CREB (Figures 2(c) and 2(d)). This suggested that nuclear translocation of the BDNF/TrkB axis is closely related to axonal malnutrition in mice with Alzheimer-like phenotypes.

Autophagosome contains APP, A*β* and its precursor *β*-C-terminal fragment, APP-metabolized intermediate cleavage enzyme, *β* secretase, and *γ* secretase, suggesting that A*β* might be generated in autophagosomes [29]. Autophagy is one of the main ways for intracellular A*β* to be eliminated, as A*β* produced by autophagosomes would be rapidly transported into lysosome via autophagosome and be degraded by cathepsin under normal circumstances [30]. However, if there are disorders during fusion and maturation of autophagosomes and lysosomes as or during degradation of contents, A*β* would be accumulated due to the failure of efficient degradation under pathological circumstances. At the late stage of AD progression, a large amount of autophagosomes and immature autophagosomes would be abnormally gathered in neuronal processes of cortex and hippocampus regions in AD patients and mice with Alzheimer-like phenotypes, suggesting the disorder occurs during fusion and maturation of autophagosomes and lysosomes at the advanced stage of AD [31]. At that time, to activate autophagy will aggravate accumulation of autophagosomes, creating an environment favorable for A*β* deposition [32].

Therefore, dual fluorescence mRFP-GFP-LC3 system was used for cell transfection in this study. LC3 transports RFP and GFP to be largely accumulated in autophagosome precursors and membranes. As a result, yellow dots under a fluorescence microscope can be seen. In an autolysosome acidic environment induced by autophagosome-lysosome fusion, LC3-carrying GFP undergoes quenching so that RFP is difficult to be degraded and autolysosome presents as a red dot. Therefore, mRFP can mark and trace LC during the whole course while weakened GFP signal suggests autophagosome-lysosome fusion. To validate the effect of autolysosome formation on BDNF nuclear translocation in AD *in vitro*, mouse hippocampal neuron HT22 cells were treated with the proton pump inhibitor Bafilomycin A1 of different concentrations (10 nM, 30 nM, and 50 nM) for 24 h to inhibit the autolysosome formation. Immunofluorescence staining results revealed that as the concentration of Bafilomycin A1 intervention increased, lysosome fusion with autophagosome was inhibited and BDNF intranuclear expression was reduced, presenting dose dependence. Then, in subsequent experiments, we only used 30 nM Bafilomycin A1 to treat the cells (Figures 2(e) and 2(f)).

In addition, HT22 cell apoptosis was promoted upon the increase of Bafilomycin A1 concentrations (Supplementary Figure 1). Microscopic observation under an inverted microscope revealed that HT22 cells treated with 30 nM Bafilomycin A1 had significantly shorter axons and smaller cell bodies compared with that of the untreated group (control group) (Figure 2(g)). Western blot analysis results showed that nuclear expression of BDNF and ratio of p-TrkB/TrkB and p-CREB/CREB were significantly reduced in cells treated with 30 nM Bafilomycin A1 for 24 h (Figure 2(h)).

All these data suggested that the autolysosome formation could improve NAD in AD by promoting BDNF nuclear translocation.

### 3.3. TRPML1 Overexpression Promotes Autolysosome-Lysosome Fusion and Facilitates BDNF Nuclear Transport to Block NAD in AD

TRPML1 is of importance to signal transduction, lysosomal ion homeostasis maintenance, membrane transportation, and exocytosis [33]. In APP/PS1 mouse model, TRPML1 has been reported to alleviate cognitive impairment in AD by mediating neuron autophagy and reducing autophagosome accumulation through the PPAR*γ*/AMPK/mTOR signaling pathway [13]. Thus, it was inferred that TRPML1 could be regarded as a novel therapeutic target for axonopathy of mice with Alzheimer-like phenotypes by promoting BDNF nuclear translocation through mediating centripetal movement of lysosomes and autophagosome-lysosome fusion.

Next, to examine the expression of TRPML1 in HT22 cells after oe-TRPML1 treatment, primary neurons from brain tissues of APP/PS1 mice were isolated. Cellular immunohistochemical experiments were employed to detect the localization in HT22 cells after overexpression of TRPML1. The results demonstrated an enhanced TRPML1 expression in the cytoplasm and nuclear periphery of HT22 cells after transfection with oe-TRPML1 (Supplementary Figure 2) [34]. Immunofluorescence staining results further revealed colocalization of TRPML1 and the lysosome marker LAMP1, suggesting a close association between TRPML1 and lysosome formation (Supplementary Figure 3).

**Figure 3:**
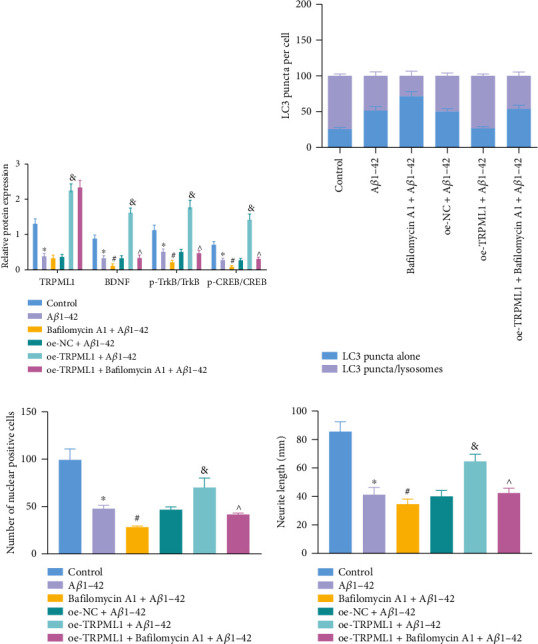
Overexpression of TRPML1 promotes autophagosome-lysosome fusion and stimulates BDNF nuclear transport to block NAD in AD. (a) Protein expression of BDNF and the ratio of p-TrkB/TrkB and p-CREB/CREB in neurons, evaluated by Western blot analysis. (b) Changes of autolysosome formation in neurons identified by immunofluorescence staining. (c) Changes of BDNF nuclear translocation detected by immunofluorescence staining. (d) Length of axons in HT22 cells observed under an inverted microscope. Measurement data were described as the mean ± standard deviation. One-way ANOVA was used for comparisons among multiple groups, followed by Tukey's post hoc test. The cell experiment was repeated 3 times independently. ^∗^*p* < 0.05 vs. untreated HT22 cells; ^#^*p* < 0.05 vs. cells treated with A*β*1-42; ^&^*p* < 0.05 vs. cells treated with oe-NC+A*β*1-42. ^^^*p* < 0.05 vs. cells treated with oe-TRPML1+A*β*1-42.

Additionally, the advanced stage of autophagy is comprised of autophagosome-lysosome fusion and autolysis acidification, necessary to maintain functional autophagy flux and cell homeostasis, both of which can be disturbed by autophagy inhibitor Bafilomycin A1 [35]. To further validate the relationship between TRPML1 and autolysosome formation in AD progression, TRPML1 was overexpressed in A*β*1-42-treated HT22 cells treated with or without autophagy inhibitor Bafilomycin A1. As the results of Western blot analysis revealed, the p-TrkB/TrkB and p-CREB/CREB ratios and the BDNF nuclear translocation were suppressed in A*β*1-42-treated neuron relative controls while treatment of overexpressed TRPML1 reversed the aforementioned results. After Bafilomycin A1 exposure to A*β*1-42-treated neurons, expression of TRPML1 did not differ, while decreased nuclear translocation of BDNF and ratios of p-TrkB/TrkB and p-CREB/CREB were observed compared to untreated HT22 cells. We also found that oe-TRPML1 rescued A*β*1-42-treated neurons from the effects of Bafilomycin A1, as evidenced by no difference concerning the expression of TRPML1 along with significantly reduced expression of BDNF, and ratio of p-TrkB/TrkB and p-CREB/CREB, as well as BDNF nuclear translocation (Figure 3(a)).

Furthermore, the formation of autolysosomes was evaluated by tandem fluorescent mRFP-GFP-LC3 assay, and the results showed A*β*1-42 reduced the formation of autolysosomes. The overexpression of TRPML1 resulted in increased autolysosomes in HT22 cells with A*β*1-42 stimulation. Stimulation with Bafilomycin A1 inhibited the formation of autolysosomes in HT22 cells with A*β*1-42 induction. In addition, less formation of autolysosomes was noted in HT22 cells with oe-TRPML1+Bafilomycin A1+A*β*1-42 than with oe-TRPML1+A*β*1-42 (Figure 3(b)). As immunofluorescence staining results revealed, the BDNF intranuclear expression was reduced in the cells with A*β*1-42 stimulation while it was elevated following overexpression of TRPML1. Additional Bafilomycin A1 counterweighed the action of TRPML1 overexpression. In addition, BDNF intranuclear expression was lower in HT22 cells with oe-TRPML1+Bafilomycin A1+A*β*1-42 than with oe-TRPML1+A*β*1-42 (Figure 3(c)).

Furthermore, observation results under an inverted microscope for cellular morphology revealed that TRPML1 overexpression rescued the shortened axons and reduced cell bodies caused by A*β*1-42, while Bafilomycin A1 further shortened axons and reduced cell bodies compared with A*β*1-42 treatment alone (Figure 3(d)).

As expected, cotreatment of oe-TRPML1 and Bafilomycin A1 in A*β*1-42-treated HT22 cells induced shorter axon and smaller cell bodies compared with that of A*β*1-42-treated HT22 cells with oe-TRPML1 alone. These results suggested that TRPML1 overexpression can increase perinuclear distribution and can promote lysosome fusion with autophagosomes to ameliorate NAD in AD.

### 3.4. TRPML1 Facilitates Autophagosome-Lysosome Fusion through p62-Mediated Recruitment of Dynein

TRPML1 promotes centripetal movement of lysosome from cell bodies towards perinuclear area by mediating dynein via lysosomal Ca^2+^ effector in favor of efficient fusion of perinuclear autophagosome, hence contributing to cell growth and metabolism [36]. The activation of dynein requires a ternary complex with its activator and adaptor proteins to recognize continuous one-way transportation parallel to microtubule orbit [37].

**Figure 4:**
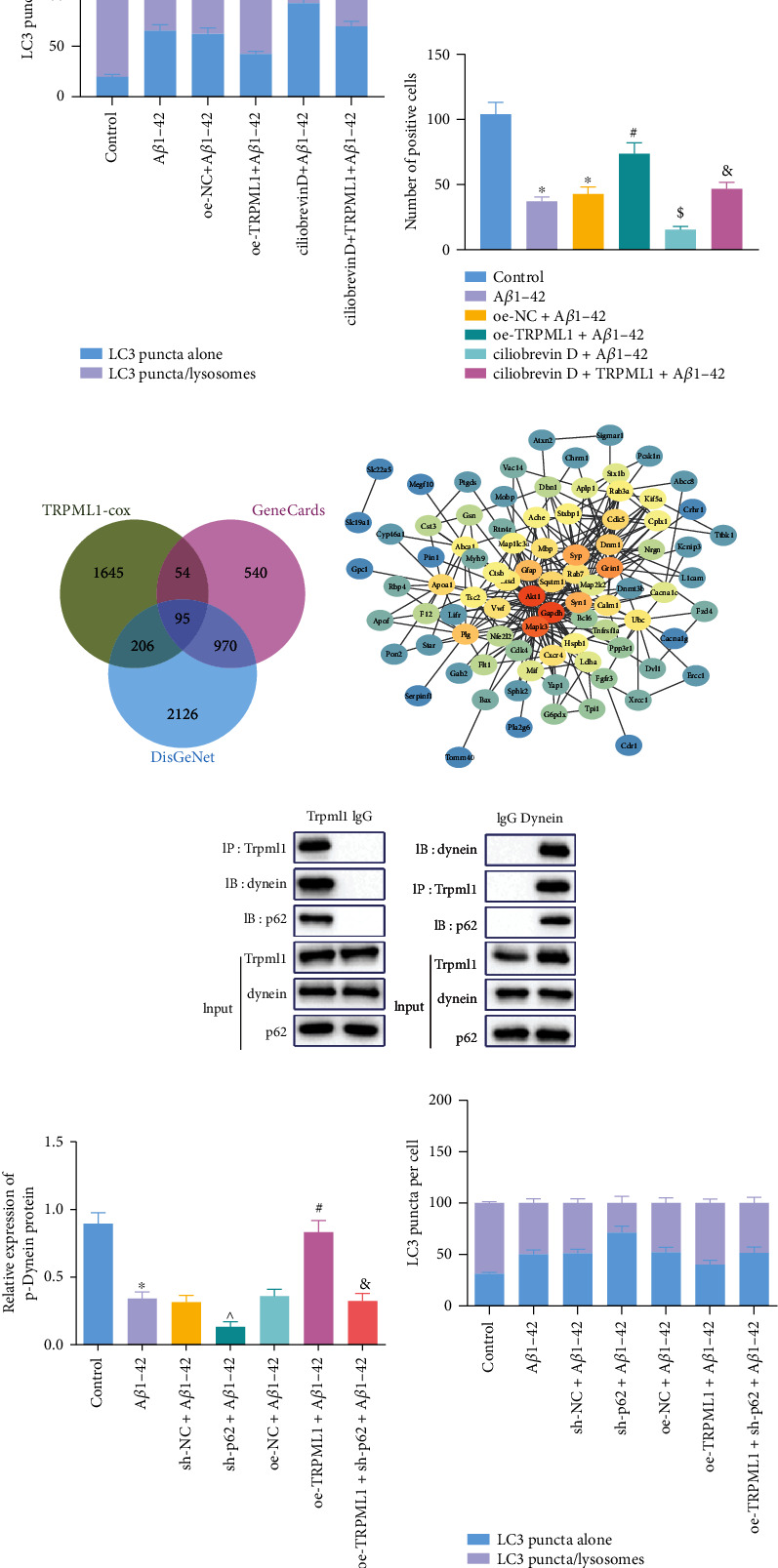
TRPML1 facilitates autophagosome-lysosome fusion through p62-mediated recruitment of dynein. (a) Changes of autolysosome formation in neurons detected by immunofluorescence staining. (b) Nuclear expression of BDNF in mouse hippocampal neurons identified by immunofluorescence staining. (c) Venn diagram flagging the intersection of TRPML1-related genes and AD-related genes in the GeneCards database and DisGeNet database. (d) Interaction network diagrams of the candidate genes, wherein the circle color from blue to orange represents the gene degree from small to large. (e) The interaction between TRPML1, p62, and dynein, confirmed by Co-IP. Neurons were treated with sh-p62 and/or oe-TRPML1 in the presence of A*β*1-42. (f) The extent of dynein phosphorylation in neurons. (g) Fusion of lysosomes and autophagosomes in neurons detected by immunofluorescence staining. Measurement data were described as the mean ± standard deviation. One-way ANOVA was used for comparisons among multiple groups, followed by Tukey's post hoc test. The cell experiment was repeated 3 times independently. ^∗^*p* < 0.05 vs. untreated HT22 cells; ^#^*p* < 0.05 vs. cells treated with oe-NC+A*β*1-42; ^^^*p* < 0.05 vs. cells treated with sh-NC+A*β*1-42 or oe-TRPML1+A*β*1-42; ^&^*p* < 0.05 vs. cells treated with oe-TRPML1+A*β*1-42 or ciliobrevin D+A*β*1-42.

In order to further investigate whether the autolysosome formation regulated by TRPML1 is associated with recruitment of dynein, primary neurons were isolated from brain tissues of APP/PS1 mice, transfected with overexpressed TRPML1, and treated with dynein inhibitor ciliobrevin D. The autolysosome formation of cells was detected by mRFP-GFP-LC3. It was observed that A*β*1-42-treated cells exhibited an inhibited formation of autolysosomes. Overexpressing TRPML1 increased the formation of autolysosomes, whereas ciliobrevin D alone or combined with TRPML1 overexpression led to a decreased formation of autolysosomes in A*β*1-42-treated cells (Figure 4(a)). Furthermore, immunofluorescence staining results revealed that BDNF intranuclear expression was reduced in A*β*1-42-treated cells while the intranuclear expression was elevated following overexpression of TRPML1. Yet, ciliobrevin D alone or combined with TRPML1 overexpression led to a decrease in the BDNF intranuclear expression in A*β*1-42-treated cells (Figure 4(b)). All in all, these results demonstrated that TRPML1 promotes autolysosome formation through dynein recruitment.

For further investigation on the underlying mechanism, we obtained the correlation of TRPML1 with other genes based on GSE5281 and used the jvenn tool to take the intersection of the top 2000 TRPML1-related genes with the smallest *p* value and AD-related genes to obtain 99 genes (Figure 4(c)). We obtained the interaction network between candidate genes by utilizing the STRING tool (Figure 4(d)) and found that the degree of 12 genes, AKT1, GAPDH, MAPK3, GRIN1, SYP, SYN1, GFAP, PLG, DNM1, APOA1, CDK5, and SQSTM1 (Alias: p65) was higher. Among them, it is well documented that the accumulation of p62/SQSTM1 and LC3-II could be inhibited by TRPML1 stimulation in motoneurons exposed to the toxin [38].

Co-IP results showed the presence of dynein and p62 expression in the TRPML1-antibody immunoprecipitation, and the presence of TRPML1 and p62 expression in the dynein-antibody immunoprecipitation (Figure 4(e)). It suggested an interaction relationship among TRPML1, p62, and dynein, suggesting that TRPML1, p62, and dynein were located in the same protein complex.

It has been reported that p62, a junction protein, could provide phosphorylation scaffolds for dynein to participate in dynein activation [16]. We thus speculated that TRPML1 may regulate autolysosome formation through p62-mediated recruitment of dynein to improve NAD in mice with Alzheimer-like phenotypes. For verification purposes, primary neurons were isolated from brain tissues of APP/PS1 mice. Additionally, TRPML1 was overexpressed and/or p62 was inhibited in cells following A*β*1-42 treatment. The phosphorylation extent of dynein was then determined by Western blot analysis, which was found to be downregulated by A*β*1-42 without significant difference regarding total dynein protein expression, while the extent of dynein phosphorylation was restored by oe-TRPML1 compared with that of the control. In contrast, sh-p62 alone or combined with oe-TRPML1 reduced the phosphorylation extent of dynein in cells (Figure 4(f)).

For autolysosome formation, overexpressing TRPML1 induced autolysosome formation and sh-p62 alone or combined with oe-TRPML1 treatment reversed the effect. AS suggested by immunofluorescence staining, BDNF intranuclear expression was elevated following treatment of overexpressed TRPML1 in the HT22 cells with A*β*1-42 induction; yet, additional delivery of sh-p62 alone or combined with oe-TRPML1 counterweighed the action of TRPML1 overexpression (Figure 4(g)).

The results confirmed that TRPML1 recruited dynein by interacting with p62 to promote autophagosome-lysosome fusion.

### 3.5. TRPML1/p62 Mediates BDNF/TrkB Signaling Pathway and Axonal Development In Vitro

**Figure 5:**
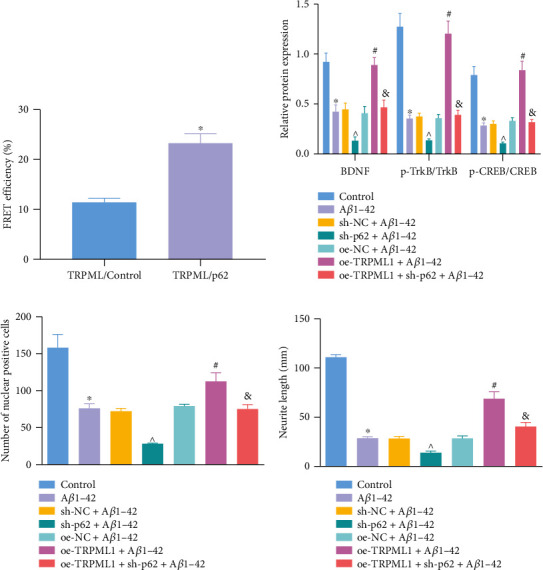
TRPML1/p62 mediates the BDNF/TrkB signaling pathway and axonal development *in vitro*. (a) Interaction between TRPML1 and p62 assessed by FRET. (b) Protein expression of BDNF and the ratio of p-TrkB/TrkB and p-CREB/CREB in neurons, evaluated by Western blot analysis. (c) BDNF nuclear translocation in neurons with alternative transfection detected by immunofluorescence staining. (d) Length of axons in neurons observed under an inverted microscope. Measurement data were described as the mean ± standard deviation. One-way ANOVA was used for comparisons among multiple groups, followed by Tukey's post hoc test. The cell experiment was repeated 3 times independently. ^∗^*p* < 0.05 vs. untreated HT22 cells; ^^^*p* < 0.05 vs. cells treated with sh-NC; ^#^*p* < 0.05 vs. cells treated with oe-NC; ^&^*p* < 0.05 vs. cells treated with oe-TRPML1.

In order to clarify the interactions between TRPML1 and p62, we verified the direct interactions between TRPML1 and p62 *in vivo* using the FRET technique. After simultaneous injection of p62-C1-YFP and pCFP-TRPML1, the yellow fluorescence signal at about 545 nm was significantly enhanced when excited by 458 nm laser (Figure 5(a)), suggesting an interaction between TRPML1 and p62 *in vivo*.

Western blot analysis and immunofluorescence staining results showed A*β*1-42 treatment resulted in reduced expression of BDNF and ratio of p-TrkB/TrkB and p-CREB/CREB as well as the intranuclear expression of BDNF, which were reversed when p62 was silenced or TRPML1 was overexpressed. The expression of BDNF and ratio of p-TrkB/TrkB and p-CREB/CREB, as well as the intranuclear expression of BDNF in A*β*1-42-treated cells with TRPML1 overexpression and p62 knockdown, were decreased, compared with that of A*β*1-42-treated cells in the presence of overexpressed TRPML1 alone (Figures 5(b) and 5(c)).

Additionally, immunofluorescence was used to observe the changes of axon under a microscope. As shown in Figure 5(d), A*β*1-42 led to shorter axon length and smaller cell bodies. sh-p62 alone resulted in reduced axon length and cell bodies while oe-TRPML1 treatment alone induced opposite changes. Compared with oe-TRPML1 treatment alone, the axon length and cell bodies were reduced in the cells upon A*β*1-42 stimulation with TRPML1 overexpression and p62 knockdown.

These results suggested that TRPML1 alleviated NAD in AD by interacting with p62 and promoting BDNF nuclear translocation.

### 3.6. Overexpressing TRPML1 Protects Axons by Interacting with p62 in APP/PS1 Transgenic Mice

**Figure 6:**
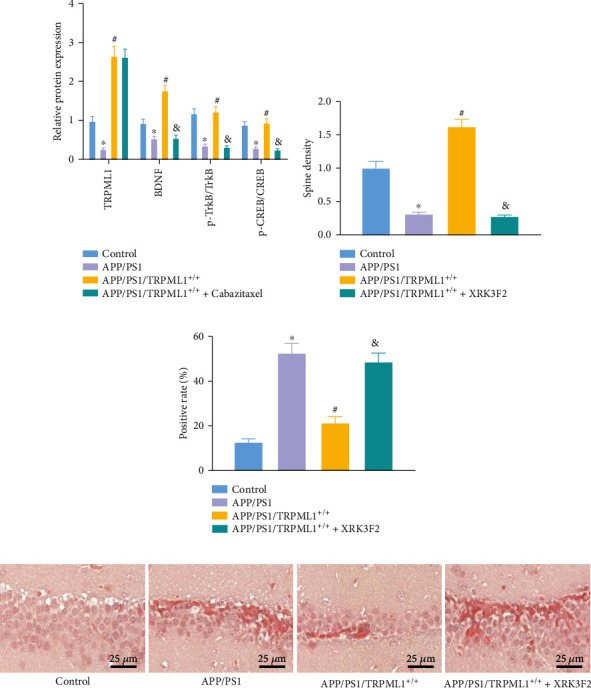
Overexpression of TRPML1 protects axons by interacting with p62 in APP/PS1 transgenic mice. (a) Protein expression of BDNF and the ratio of p-TrkB/TrkB and p-CREB/CREB in hippocampal tissues of mice evaluated by Western blot analysis. (b) Dendrites and dendritic spines in hippocampal tissues of mice evaluated by Golgi-Cox staining. (c) Cell apoptosis in hippocampal tissues of mice, tested by TUNEL assay. (d) Senile plaques in hippocampal tissues of mice, evaluated by Congo red staining. Measurement data were described as the mean ± standard deviation. One-way ANOVA was used for comparisons among multiple groups, followed by Tukey's post hoc test. *n* = 12 for mice in each group. ^∗^*p* < 0.05 vs. C57BL/6J WT mice; ^#^*p* < 0.05 vs. APP/PS1 mice; ^&^*p* < 0.05 vs. APP/PS1/TRPML1^+/+^ mice.

To assess whether p62 is involved in TRPML1-medicated axonal development in AD, APP/PS1/TRPML1^+/+^ transgenic AD male mice were treated with p62 inhibitor XRK3F2. In the hippocampal tissues, the expression of TRPML1 and BDNF proteins and the ratios of p-TrkB/TrkB and p-CREB/CREB were then evaluated using Western blot analysis (Figure 6(a)). We found declines in the TRPML1 and BDNF expression and p-TrkB/TrkB and p-CREB/CREB ratios in the hippocampal tissues of APP/PS1 mice than those in WT C57BL/6J mice. APP/PS1/TRPML1^+/+^ mice exhibited upregulated TRPML1 and BDNF expression and increased p-TrkB/TrkB and p-CREB/CREB ratios. Further treatment with XRK3F2 decreased the expression of BDNF, and p-TrkB/TrkB and p-CREB/CREB ratios in APP/PS1/TRPML1^+/+^ mice without altering the expression of TRPML1.

Then, as observed by Golgi-cox staining, the APP/PS1 mice presented with reduced neuronal dendritic spines relative to the control mice, which were increased in the APP/PS1/TRPML1^+/+^ mice and decreased in the APP/PS1/TRPML1^+/+^+XRK3F2 mice when compared with that of APP/PS1 mice (Figure 6(b)). Furthermore, neuronal apoptosis affected by TRPML1 and p62 in APP/PS1 mice was detected by TUNEL staining. The neuronal apoptosis was found to be decreased in APP/PS1/TRPML1^+/+^ mice than that in APP/PS1 mice, while it was enhanced following further treatment with XRK3F2 (Figure 6(c)). Finally, the tangles of nerve fibers were detected by Congo red staining. The results showed that the entanglement of nerve fibers and senile plaques in the hippocampal tissues of APP/PS1 mice was significantly elevated, and meanwhile, consistent results were noted in APP/PS1/TRPML1^+/+^ mice in the presence of XRK3F2 (Figure 6(d)).

In summary, these data suggested that TRPML1 could attenuate NAD in APP/PS1 transgenic mice by interacting with p62.

## 4. Discussion

The TRPML1 antagonist has been established to block low-density lipoprotein-induced enhancements in intraneuronal and secreted expression of A*β* along with A*β* accumulation in endolysosomes [39]. It has been validated that AD pathology is strongly linked to mitochondrial dysfunction induced by A*β* and BDNF axonal transport deficits [40]. Swollen regions of axons and dendrites with autophagy-associated organelles are a highly characteristic and widespread form of NAD in AD, indicating dysfunction of autophagy and axonal transport [41]. The purpose of our work is to confirm the influences of TRPML1 on NAD in AD, with the underlying molecular mechanisms investigated.

It was reported that TRPML1 is poorly expressed, and the BDNF/TrkB signaling pathway and BDNF nuclear translocation are blocked, all of which are associated with NAD in AD. Moreover, overexpressing TRPML1 improved the memory and recognition indexes and diminished neuronal apoptosis in the APP/PS1 transgenic mice [13]. BDNF, the most extensively distributed neurotrophins in the adult brain, exerts a neurotrophic protection on neurons by interacting with its specific receptor, TrkB [42]. Intracerebral injection of AM206, the BDNF antagomir, enhances memory function, synaptic density, and neurogenesis in Tg2576 mice [43]. This suggested that TRPML1 played neuroprotective roles in AD by restoring the BDNF nuclear translocation in the neurons. Our results demonstrated an obviously downregulated TRPML1 expression in the APP/PS1 double transgenic mice and AD cells and overexpressing TRPML1 induced autolysosome formation, nuclear translocation of BDNF, and BDNF/TrkB signaling pathway activation. TRPML1 induction near the plasma membrane may stimulate lysosomal exocytosis [44, 45], whereas perinuclear TRPML1 lysosomes trigger autolysosome formation [33]. In addition, Jin et al. displayed that BDNF enhanced autophagy flux, and BDNF/TrkB-governed mitophagy protects brain microvascular endothelial cells from injury caused by high levels of glucose [46]. Bordi et al. demonstrated that continued promotion of autophagy is a response to reducing lysosomal clearance of substrates, which explains the exceptionally robust autophagic pathology and NAD that is associated with AD pathogenesis [47]. In this study, we revealed that repressed autolysosome formation blocked the BDNF nuclear translocation in the mouse hippocampal region. These results suggested that the TRPML1 exerts neuroprotective effects against AD by restoring autolysosome formation involved in the activated BDNF/TrkB regulatory axis and the p62-dependent dynein.

Our findings also demonstrated that TRPML1 is engaged in the regulation of autolysosome formation in AD cells and mouse models through p62-mediated recruitment of dynein. A previous study indicated that cytoplasmic dynein links to dynactin and cargo adaptor proteins to produce a transport mechanism equipped with capabilities of long-distance processive movement along microtubules [37]. Kimura et al. confirmed that aging inhibits the communication between dynein-dynactin complexes in the brain of cynomolgus monkey and dynein dysfunction led to subsequent endocytic disturbances, intracellular A*β*1 accumulation, deficiencies of synaptic vesicle transport, and neurotic swelling [48]. More intriguingly, TRPML1 inactivation has been discovered to lead to the accumulation of lysosomal tau, indicating that TFEB-TRPML1 signaling may function as an innate clearance mechanism to eliminate aberrant intracellular tau through lysosomal exocytosis of seeding-incompetent tau [49]. The activation of the TRPML1/ALG-2/dynein signaling axis has been implicated to promote minus-end-directed motion of lysosomes, which contributes to quick redistribution of lysosomes to the juxtanuclear region, hence facilitating autolysosome formation [36]. Additionally, dynein knockdown promotes p62 expression and the accumulation of autophagic vacuoles, while blocking the autophagosome fusion with lysosome [50]. More specifically, BDNF may inhibit the autophagy induced by 3-nitropropionic acid, which further protects the primary cortical neurons through upregulation of p62, whereas downregulation of p62 abolishes BDNF-dependent neuroprotection [51]. The inhibition of p62 by shRNA *in vitro* and XRK3F2 *in vivo* suppressed potentiated BDNF nuclear translocation induced by TRPML1 overexpression, an outcome that has been consistent in this study. Snapin, a dynein adaptor, functions by regulating retrograde axonal transport of the TrkB signaling pathway [52] by binding to its intermediate chain. Most recently, Olenick et al. also demonstrated that Hook1, an effector of dynein, could regulate the BDNF-TrkB axis endosome transport in primary neurons in the hippocampal region [53]. Of note, RNA interference is widely applied for inhibition of genes, yet the approach is prone to off-target effects. Besides, it has been proposed clustered regularly interspaced short palindromic repeats/cas9 method as an alternative approach through genetic mutation, and scrambled shRNAs have also stood out for gene knockdown investigation from cellular level to animal behavior [54, 55].

## 5. Conclusions

**Figure 7:**
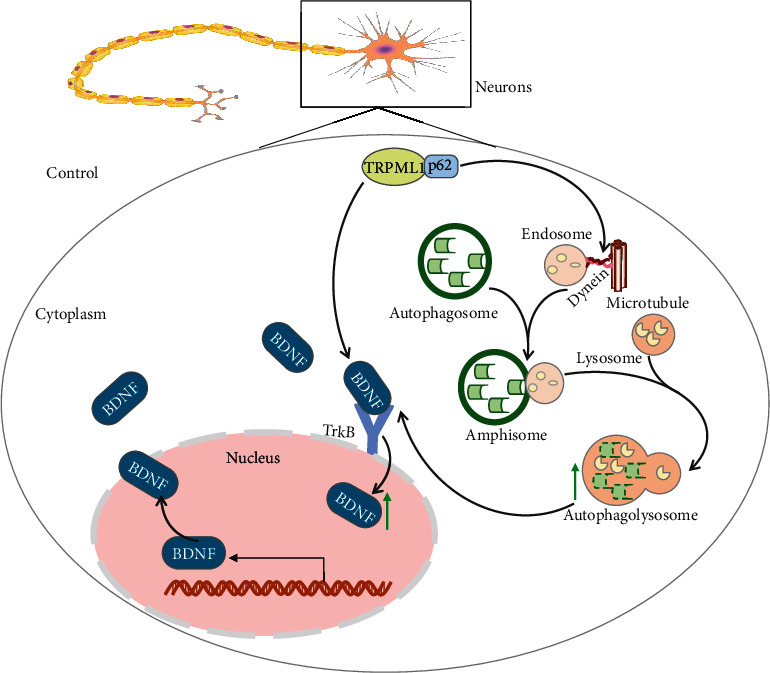
A schematic diagram illustrating the NAD regulatory mechanism of TRPML1 in AD. TRPML1 promotes the autophagosome-lysosome fusion through recruitment of dynein by interacting with p62 and stimulates the nuclear translocation of BDNF, ultimately attenuating the NAD in AD.

In summary, we demonstrated that overexpression of TRPML1 played a neuroprotective role in AD by improving cognitive function, preventing the cognitive impairments, and alleviating neuron apoptosis. Moreover, TRPML1 activated the BDNF/TrkB signaling pathway and interacted with the p62-dependent dynein to attenuate NAD in AD (Figure 7), providing new insights into understanding pathology and potential therapeutic targets for AD. Since limited evidence exists regarding the role of TRPML1 in axonal transport, future studies to understand the mechanistic link between TRPML1 and axonal transport are needed. Besides, additional studies are warranted to solve the limitation of this study and determine the mechanistic actions of TRPML1 on autophagy based on the detection of the activity and expression of other common autophagy proteins, such as LC3, Beclin-1, ULK1, Atg5, and Atg7.

## Data Availability

The datasets generated/analyzed during the current study are available from the corresponding author upon reasonable request.

## Supplementary Materials

Supplementary Materials Supplementary Figure 1: HT22 cell apoptosis under different concentrations of Bafilomycin A1 (0-50 *μ*M) detected by flow cytometry. Measurement data were described as the mean ± standard deviation. An unpaired *t*-test was used for comparison between the two groups. The cell experiment was repeated 3 times independently. ^∗^*p* < 0.05 vs. 0 *μ*M Bafilomycin A1 treatment. Supplementary Figure 2: TRPML1 expression in the HT22 cells detected by immunohistochemistry and RT-qPCR. Measurement data were described as the mean ± standard deviation. An unpaired *t*-test was used for comparison between the two groups. The cell experiment was repeated 3 times independently. ^∗^*p* < 0.05 vs. cells treated with oe-NC. Supplementary Figure 3: TRPML1 is colocalized with lysosome marker LAMP1 identified by immunofluorescence staining. The cell experiment was repeated 3 times independently.
