# Supplementary material for: Interaction between TRPML1 and p62 in Regulating Autophagosome-Lysosome Fusion and Impeding Neuroaxonal Dystrophy in Alzheimer's Disease
Source: Oxid Med Cell Longev. 2022 Jan 25;2022:8096009. doi: 10.1155/2022/8096009 (PMC8807035; doi:10.1155/2022/8096009)
Supplement: Supplementary Materials — Supplementary Figure 1: HT22 cell apoptosis under different concentrations of Bafilomycin A1 (0-50 μM) detected by flow cytometry. Measurement data were described as the mean ± standard deviation. An unpaired t-test was used for comparison between the two groups. The cell experiment was repeated 3 times independently. ∗p < 0.05 vs. 0 μM Bafilomycin A1 treatment. Supplementary Figure 2: TRPML1 expression in the HT22 cells detected by immunohistochemistry and RT-qPCR. Measurement data were described as the mean ± standard deviation. An unpaired t-test was used for comparison between the two groups. The cell experiment was repeated 3 times independently. ∗p < 0.05 vs. cells treated with oe-NC. Supplementary Figure 3: TRPML1 is colocalized with lysosome marker LAMP1 identified by immunofluorescence staining. The cell experiment was repeated 3 times independently. [file 8096009.f1.zip › Table S1 (1).docx]

**Table S1** Primer sequences for RT-qPCR

| Target gene | Primer sequence |
| --- | --- |
| BDNF | F: 5’-AGAGCTGTTGGATGAGGACCAG-3’ |
|  | R: 5’-CAAAGGCACTTGACTACTGAGCA-3’ |
| TRPML1 | F: 5’-CAAGATCTTGGTGGTCACTGTGCAG-3’ |
|  | R: 5’-GGTTGCTGAGCCCAAAGAGAATGAG-3’ |
| TrkB | F: 5’-GTCTGGAGGGTGCTATGCTA-3’ |
|  | R: 5’-CAGGGGCAGAAACTCCAGAA-3’ |
| CREB | F: 5’-TGTACCACCGGTATCCATGC-3’ |
|  | R: 5’-TGGATAACGCCATGGACCTG-3’ |
| GAPDH | F: 5’-TTCACCACCATGGAGAAGGC-3’ |
|  | R: 5’-GGCATGGACTGTGGTCATGA-3’ |

Note: RT-qPCR, reverse transcription quantitative polymerase chain reaction; F, forward; R, reverse; BDNF, brain-derived neurotrophic factor; TRPML1, transient receptor potential mucolipin 1; TrkB, tyrosine kinase receptor B; CREB, cAMP-response element binding protein; GAPDH, glyceraldehyde-3-phosphate dehydrogenase.
