# Supplementary figures and images for: Interaction between TRPML1 and p62 in Regulating Autophagosome-Lysosome Fusion and Impeding Neuroaxonal Dystrophy in Alzheimer's Disease
Source: Oxid Med Cell Longev. 2022 Jan 25;2022:8096009. doi: 10.1155/2022/8096009 (PMC8807035; doi:10.1155/2022/8096009)

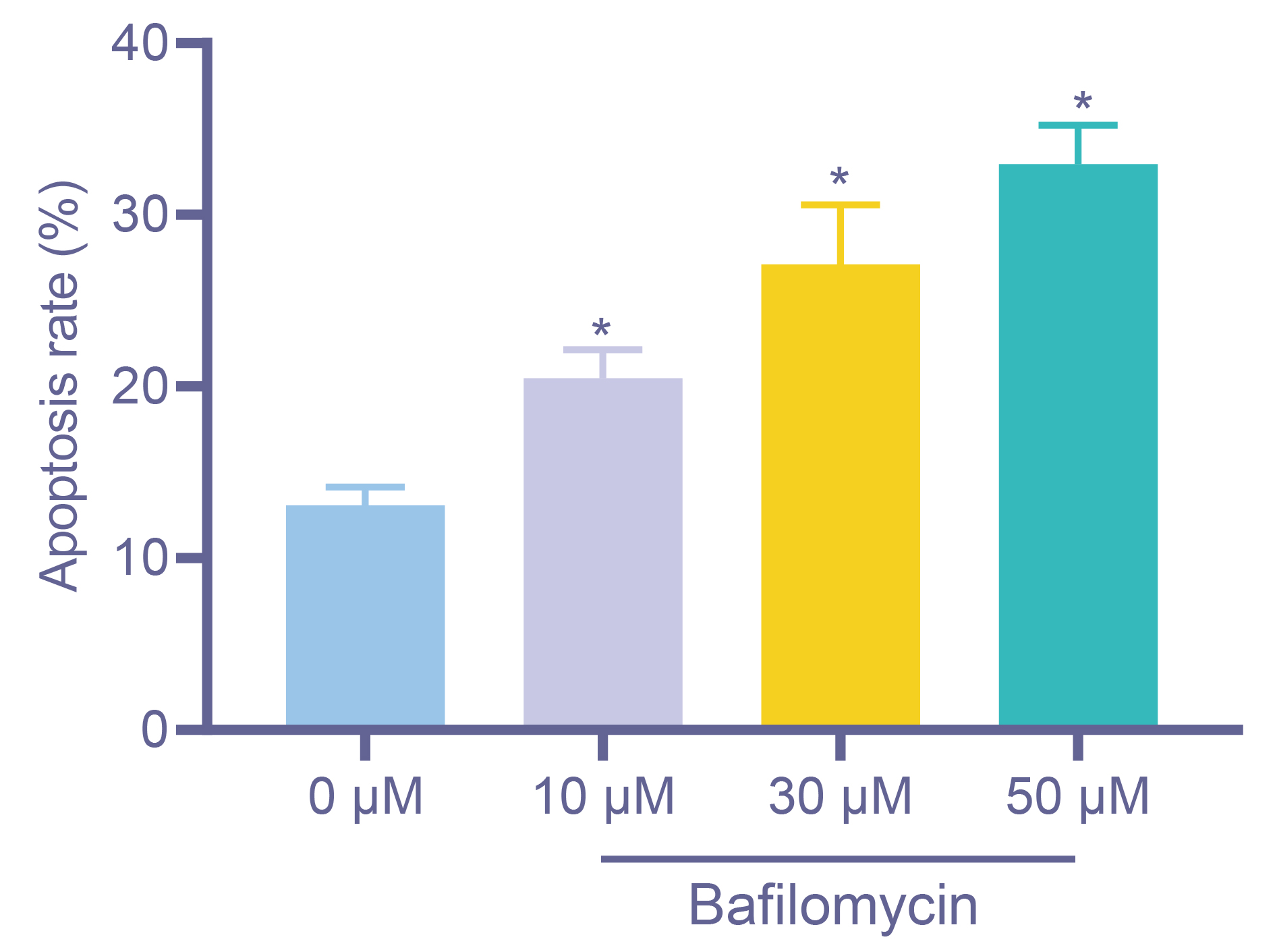

Supplement: Supplementary Materials — Supplementary Figure 1: HT22 cell apoptosis under different concentrations of Bafilomycin A1 (0-50 μM) detected by flow cytometry. Measurement data were described as the mean ± standard deviation. An unpaired t-test was used for comparison between the two groups. The cell experiment was repeated 3 times independently. ∗p < 0.05 vs. 0 μM Bafilomycin A1 treatment. Supplementary Figure 2: TRPML1 expression in the HT22 cells detected by immunohistochemistry and RT-qPCR. Measurement data were described as the mean ± standard deviation. An unpaired t-test was used for comparison between the two groups. The cell experiment was repeated 3 times independently. ∗p < 0.05 vs. cells treated with oe-NC. Supplementary Figure 3: TRPML1 is colocalized with lysosome marker LAMP1 identified by immunofluorescence staining. The cell experiment was repeated 3 times independently. [file 8096009.f1.zip › Figure S1 (1).jpg]

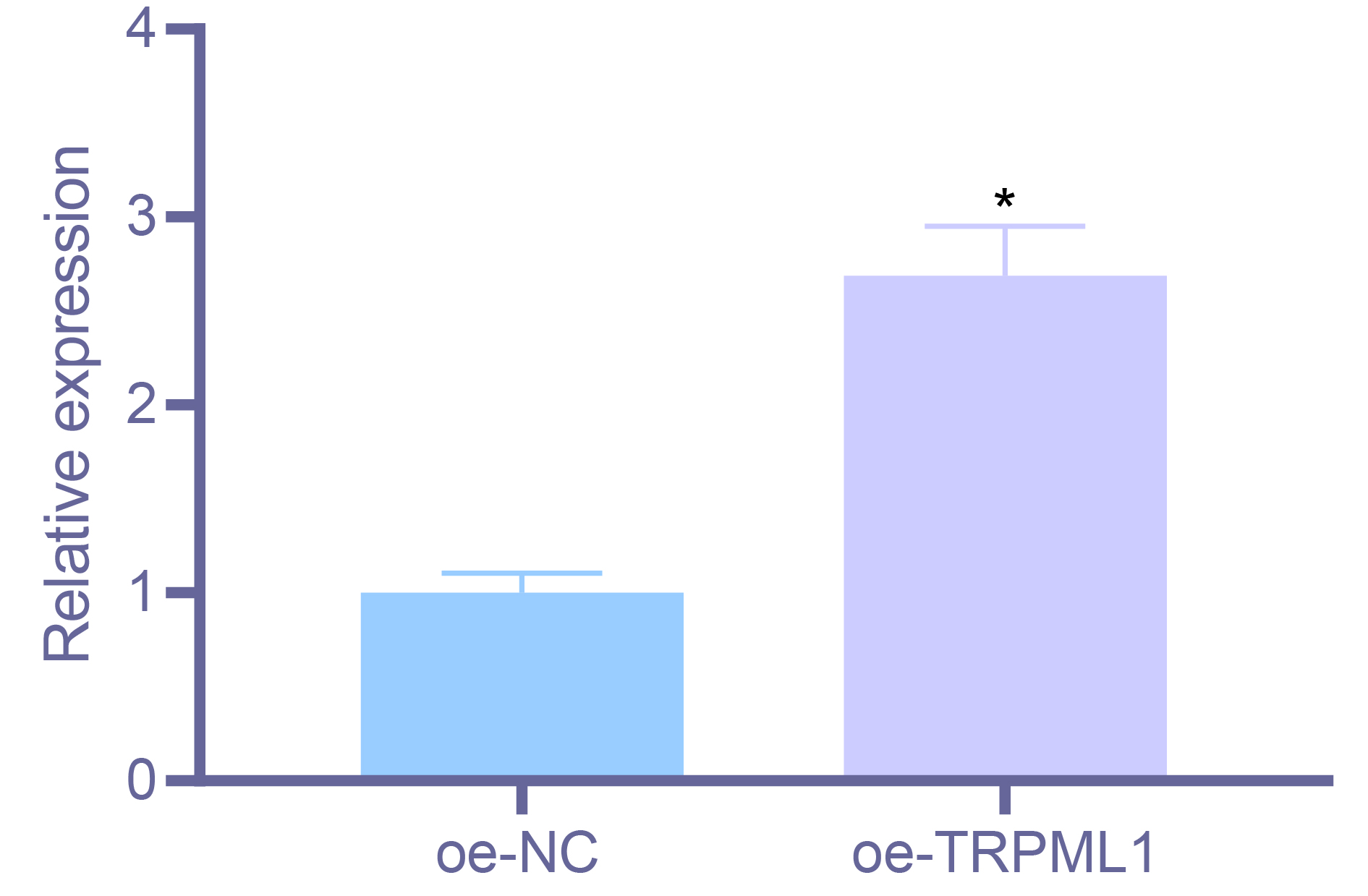

Supplement: Supplementary Materials — Supplementary Figure 1: HT22 cell apoptosis under different concentrations of Bafilomycin A1 (0-50 μM) detected by flow cytometry. Measurement data were described as the mean ± standard deviation. An unpaired t-test was used for comparison between the two groups. The cell experiment was repeated 3 times independently. ∗p < 0.05 vs. 0 μM Bafilomycin A1 treatment. Supplementary Figure 2: TRPML1 expression in the HT22 cells detected by immunohistochemistry and RT-qPCR. Measurement data were described as the mean ± standard deviation. An unpaired t-test was used for comparison between the two groups. The cell experiment was repeated 3 times independently. ∗p < 0.05 vs. cells treated with oe-NC. Supplementary Figure 3: TRPML1 is colocalized with lysosome marker LAMP1 identified by immunofluorescence staining. The cell experiment was repeated 3 times independently. [file 8096009.f1.zip › Figure S2 (1).jpg]

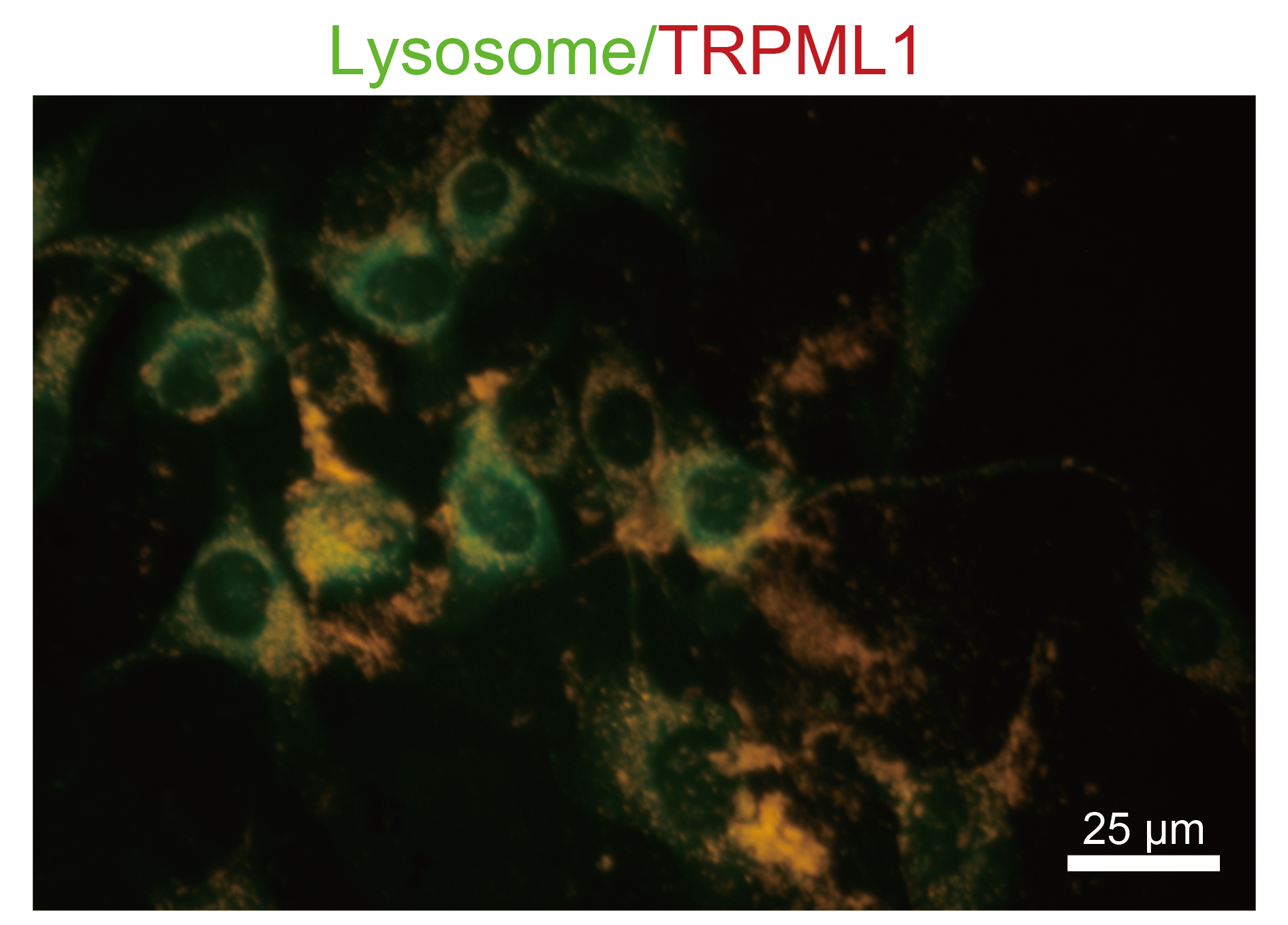

Supplement: Supplementary Materials — Supplementary Figure 1: HT22 cell apoptosis under different concentrations of Bafilomycin A1 (0-50 μM) detected by flow cytometry. Measurement data were described as the mean ± standard deviation. An unpaired t-test was used for comparison between the two groups. The cell experiment was repeated 3 times independently. ∗p < 0.05 vs. 0 μM Bafilomycin A1 treatment. Supplementary Figure 2: TRPML1 expression in the HT22 cells detected by immunohistochemistry and RT-qPCR. Measurement data were described as the mean ± standard deviation. An unpaired t-test was used for comparison between the two groups. The cell experiment was repeated 3 times independently. ∗p < 0.05 vs. cells treated with oe-NC. Supplementary Figure 3: TRPML1 is colocalized with lysosome marker LAMP1 identified by immunofluorescence staining. The cell experiment was repeated 3 times independently. [file 8096009.f1.zip › Figure S3 (1).jpg]
